# *N*-(3,4-Difluoro­phen­yl)-3,4-dimethoxy­benzene­sulfonamide

**DOI:** 10.1107/S1600536808012610

**Published:** 2008-05-03

**Authors:** Hyon Pil You, Byung Hee Han, Sung Kwon Kang, Chang Keun Sung, Sang Ook Kang

**Affiliations:** a Department of Chemistry, Chungnam National University, Daejeon 305-764, Republic of Korea; b Department of Food Science and Technology, Chungnam National University, Daejeon 305-764, Republic of Korea; c Department of Materials Chemistry, Korea University, 208 Seochang, Chochidwon, Chungnam 339-700, Republic of Korea

## Abstract

In the title sulfonamide derivative, C_14_H_13_F_2_NO_4_S, the dihedral angle between the benzene rings is 66.05 (9)°. The crystal structure is stabilized by weak inter­molecular N—H⋯O hydrogen bonds involving the amine and meth­oxy groups, which link the mol­ecules into a one-dimensional chain. No significant inter­chain contacts are observed.

## Related literature

For general background on skin-whitening agents, see: Dawley & Flurkey (1993 ▶); Nerya *et al.* (2003 ▶); Juana *et al.* (1994 ▶); Briganti *et al.* (2003 ▶). For the synthesis, see: Hussain *et al.* (2003 ▶).
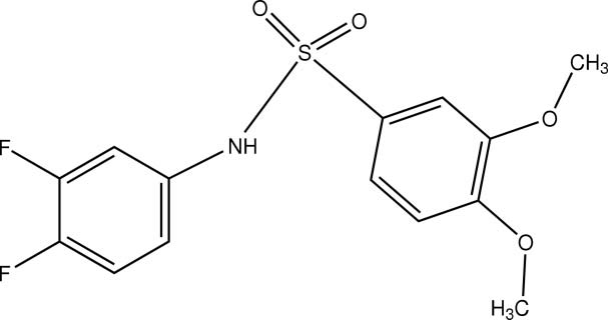


## Experimental

### 

#### Crystal data

C_14_H_13_F_2_NO_4_S
*M* *_r_* = 329.31
Monoclinic, 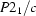

*a* = 12.2886 (10) Å
*b* = 8.5662 (7) Å
*c* = 14.5546 (12) Å
β = 109.655 (2)°
*V* = 1442.8 (2) Å^3^
*Z* = 4
Mo *K*α radiation
μ = 0.26 mm^−1^
*T* = 295 (2) K
0.25 × 0.18 × 0.15 mm

#### Data collection

Bruker SMART CCD area-detector diffractometer
Absorption correction: multi-scan (*SADABS*; Bruker, 2002 ▶) *T*_min_ = 0.928, *T*_max_ = 0.957
9690 measured reflections
3308 independent reflections
1700 reflections with *I* > 2σ(*I*)
*R*_int_ = 0.042

#### Refinement

*R*[*F* ^2^ > 2σ(*F* ^2^)] = 0.044
*wR*(*F* ^2^) = 0.126
*S* = 0.99
3308 reflections
204 parameters
H atoms treated by a mixture of independent and constrained refinement
Δρ_max_ = 0.19 e Å^−3^
Δρ_min_ = −0.21 e Å^−3^

### 

Data collection: *SMART* (Bruker, 2002 ▶); cell refinement: *SAINT* (Bruker, 2002 ▶); data reduction: *SAINT*; program(s) used to solve structure: *SHELXS97* (Sheldrick, 2008 ▶); program(s) used to refine structure: *SHELXL97* (Sheldrick, 2008 ▶); molecular graphics: *ORTEP-3 for Windows* (Farrugia, 1997 ▶); software used to prepare material for publication: *WinGX* (Farrugia, 1999 ▶).

## Supplementary Material

Crystal structure: contains datablocks global, I. DOI: 10.1107/S1600536808012610/bh2170sup1.cif

Structure factors: contains datablocks I. DOI: 10.1107/S1600536808012610/bh2170Isup2.hkl

Additional supplementary materials:  crystallographic information; 3D view; checkCIF report

## Figures and Tables

**Table 1:** Hydrogen-bond geometry (Å, °)

*D*—H⋯*A*	*D*—H	H⋯*A*	*D*⋯*A*	*D*—H⋯*A*
N7—H7⋯O17^i^	0.76 (3)	2.48 (3)	3.180 (3)	155 (3)
N7—H7⋯O19^i^	0.76 (3)	2.61 (3)	3.256 (3)	144 (3)

Symmetry code: (i) 
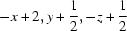
.

**Fig. 1.**
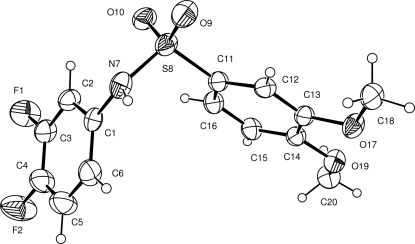
The molecular structure of the title compound, showing the atom-numbering scheme and 30% probability ellipsoids.

## supplementary crystallographic information

### Comment

Most skin whitening agents currently on the market (Dawley & Flurkey, 1993; Nerya *et al.*, 2003) contain hydroquinone, ascorbic acid, kojic acid (Juana *et al.*, 1994), arbutin, azealic acid, and glycyrrhetinic acid. They include aromatic, methoxy, hydroxyl and carbonyl functional groups in their structures. They are acting as a direct inhibitors of tyrosinase, the enzyme in the skin pigment cells (melanocytes) producing melanin.

Tyrosinase is the key enzyme converting the amino acid *L*-tyrosine to melanin, and its inhibitors are target molecules to develop anti-pigmentation agents for skin treatment after sunburn (Briganti *et al.* 2003). The melanin formation by the tyrosinase activity after sunlight exposure causes some dermatological disorders associated with freckles and melasma. Therefore, potent inhibitory agents on melanin formation and tyrosinase should be cosmetically useful for treatment of dermatological disorders.

However, most skin whitening agents have some problems, due to toxicity, low stability of formulation and poor skin permeation. In our work on the development of new whitening agents to complement the inadequacy of current whitening agents and maximize the inhibitory effects of melanin creation, we synthesized the title compound (Fig. 1), *via* a general chemical reaction (Hussain *et al.*, 2003) of 3,4-difluoroaniline with aromatic sulfonyl chloride, and studied its X-ray crystal structure.

The 3,4-dimethoxybenzenesulfonyl and 3,4-difluoroaniline moieties are essentially planar, with a mean deviation of 0.004 Å and 0.010 Å, respectively, from the corresponding least-squares planes. The dihedral angle between benzene rings is 66.05 (9)°. The intermolecular N7—H7···O17^i^ and O19^i^ [symmetry code: (i) -*x* + 2, *y* + 1/2, -*z* + 1/2] hydrogen bonds (involving the H atom of the amine and O atoms of methoxy groups) allow to form an extensive one-dimensional network along the *b* axis, which stabilizes the crystal structure.

### Experimental

3,4-difluoroaniline and 3,4-dimethoxy benzenesulfonyl chloride were purchased from Sigma Chemical Co. Solvents used for organic synthesis were redistilled before used. All other chemicals and solvents were of analytical grade and used without further purification. The title compound  was prepared by the reaction of 3,4-difluoroaniline (1 mmol) with aromatic sulfonyl chloride (1.2 mmol) in triethylamine as a solvent, under stirring. Evaporation of solvent, treatment with water, extraction with methylene chloride and chromatography of the dried solution (MgSO_4_) on silica gel column (2 / 1 = hexane / ethyl acetate) gave the title compound in 56% yield. Colourless single crystals were obtained by slow evaporation from an ethyl acetate solution, at room temperature.

### Refinement

The amine H atom H7 was located in a difference map and refined freely. The other H atoms were positioned geometrically and refined using a riding model, with C—H = 0.93 Å for aromatic H atoms and 0.96 Å for methyl H atoms, and with *U*_iso_(H) = 1.2*U*_eq_(C) for aromatic and 1.5*U*_eq_(C) for methyl H atoms.

### Crystal data

**Table:**

C_14_H_13_F_2_NO_4_S	*F*_000_ = 680
*M**_r_* = 329.31	*D*_x_ = 1.516 Mg m^−^^3^
Monoclinic, *P*2_1_/*c*	Mo *K*α radiation λ = 0.71073 Å
Hall symbol: -P 2ybc	Cell parameters from 1598 reflections
*a* = 12.2886 (10) Å	θ = 2.8–23.5º
*b* = 8.5662 (7) Å	µ = 0.26 mm^−^^1^
*c* = 14.5546 (12) Å	*T* = 295 (2) K
β = 109.655 (2)º	Block, colourless
*V* = 1442.8 (2) Å^3^	0.25 × 0.18 × 0.15 mm
*Z* = 4	

### Data collection

**Table:**

Bruker SMART CCD area-detector diffractometer	*R*_int_ = 0.042
φ and ω scans	θ_max_ = 27.5º
Absorption correction: multi-scan(SADABS; Bruker, 2002)	θ_min_ = 1.8º
*T*_min_ = 0.928, *T*_max_ = 0.957	*h* = −15→14
9690 measured reflections	*k* = −11→9
3308 independent reflections	*l* = −18→18
1700 reflections with *I* > 2σ(*I*)	

### Refinement

**Table:**

Refinement on *F*^2^	H atoms treated by a mixture of independent and constrained refinement
Least-squares matrix: full	*w* = 1/[σ^2^(*F*_o_^2^) + (0.0426*P*)^2^ + 0.5561*P*] where *P* = (*F*_o_^2^ + 2*F*_c_^2^)/3
*R*[*F*^2^ > 2σ(*F*^2^)] = 0.044	(Δ/σ)_max_ = 0.001
*wR*(*F*^2^) = 0.126	Δρ_max_ = 0.19 e Å^−^^3^
*S* = 0.99	Δρ_min_ = −0.21 e Å^−^^3^
3308 reflections	Extinction correction: SHELXL97 (Sheldrick, 2008), Fc^*^=kFc[1+0.001xFc^2^λ^3^/sin(2θ)]^-1/4^
204 parameters	Extinction coefficient: 0.0349 (19)

### Fractional atomic coordinates and isotropic or equivalent isotropic displacement parameters (Å^2^)

**Table:**

	*x*	*y*	*z*	*U*_iso_*/*U*_eq_	
F1	0.37106 (16)	0.1400 (3)	−0.04021 (13)	0.0975 (7)	
F2	0.3788 (2)	0.0318 (3)	0.13264 (17)	0.1271 (9)	
C1	0.6212 (2)	0.3457 (3)	0.11332 (19)	0.0492 (7)	
C2	0.5338 (2)	0.2966 (3)	0.0306 (2)	0.0584 (8)	
H2	0.5288	0.3352	−0.0304	0.07*	
C3	0.4554 (2)	0.1914 (4)	0.0395 (2)	0.0609 (8)	
C4	0.4593 (3)	0.1366 (4)	0.1281 (2)	0.0753 (10)	
C5	0.5430 (3)	0.1849 (5)	0.2102 (2)	0.0885 (12)	
H5	0.5454	0.1475	0.2708	0.106*	
C6	0.6248 (2)	0.2906 (4)	0.2030 (2)	0.0692 (9)	
H6	0.6826	0.3245	0.2591	0.083*	
N7	0.7042 (2)	0.4591 (3)	0.1068 (2)	0.0595 (7)	
H7	0.738 (2)	0.489 (4)	0.158 (2)	0.062 (10)*	
S8	0.78080 (6)	0.43912 (9)	0.03547 (5)	0.0595 (3)	
O9	0.84833 (17)	0.5777 (2)	0.04834 (17)	0.0802 (7)	
O10	0.70295 (15)	0.3991 (3)	−0.05866 (13)	0.0709 (6)	
C11	0.8750 (2)	0.2809 (3)	0.07756 (18)	0.0490 (7)	
C12	0.9910 (2)	0.3073 (3)	0.13211 (17)	0.0478 (6)	
H12	1.0184	0.4087	0.1468	0.057*	
C13	1.0644 (2)	0.1829 (3)	0.16380 (17)	0.0472 (6)	
C14	1.0231 (2)	0.0296 (3)	0.14189 (18)	0.0480 (6)	
C15	0.9086 (2)	0.0050 (3)	0.0875 (2)	0.0559 (7)	
H15	0.8809	−0.0962	0.0724	0.067*	
C16	0.8350 (2)	0.1300 (3)	0.0554 (2)	0.0566 (7)	
H16	0.7579	0.1128	0.0187	0.068*	
O17	1.17904 (15)	0.1936 (2)	0.21844 (13)	0.0614 (5)	
C18	1.2333 (2)	0.3419 (4)	0.2277 (2)	0.0669 (9)	
H18A	1.3129	0.3326	0.2681	0.1*	
H18B	1.1949	0.4141	0.2569	0.1*	
H18C	1.2288	0.3792	0.1643	0.1*	
O19	1.10296 (15)	−0.0832 (2)	0.17753 (14)	0.0599 (5)	
C20	1.0682 (3)	−0.2409 (3)	0.1507 (2)	0.0668 (8)	
H20A	1.132	−0.3098	0.1805	0.1*	
H20B	1.0441	−0.2514	0.0811	0.1*	
H20C	1.005	−0.2674	0.1727	0.1*	

### Atomic displacement parameters (Å^2^)

**Table:**

	*U*^11^	*U*^22^	*U*^33^	*U*^12^	*U*^13^	*U*^23^
F1	0.0780 (12)	0.1205 (18)	0.0749 (12)	−0.0470 (12)	0.0005 (10)	−0.0069 (11)
F2	0.1139 (17)	0.160 (2)	0.1037 (16)	−0.0684 (17)	0.0321 (14)	0.0185 (15)
C1	0.0374 (13)	0.0490 (16)	0.0540 (17)	0.0026 (12)	0.0060 (12)	−0.0074 (13)
C2	0.0499 (16)	0.0634 (19)	0.0522 (17)	−0.0065 (14)	0.0044 (13)	0.0047 (14)
C3	0.0480 (16)	0.069 (2)	0.0555 (18)	−0.0104 (15)	0.0037 (14)	−0.0058 (15)
C4	0.0615 (19)	0.088 (3)	0.075 (2)	−0.0210 (18)	0.0223 (17)	0.0036 (19)
C5	0.074 (2)	0.134 (4)	0.057 (2)	−0.015 (2)	0.0207 (18)	0.008 (2)
C6	0.0520 (17)	0.096 (3)	0.0530 (19)	−0.0028 (17)	0.0093 (14)	−0.0121 (17)
N7	0.0462 (13)	0.0581 (16)	0.0618 (17)	−0.0029 (12)	0.0019 (12)	−0.0102 (13)
S8	0.0460 (4)	0.0573 (5)	0.0647 (5)	−0.0070 (4)	0.0048 (3)	0.0101 (4)
O9	0.0582 (12)	0.0579 (14)	0.1098 (18)	−0.0137 (10)	0.0090 (12)	0.0198 (12)
O10	0.0543 (11)	0.0896 (17)	0.0556 (12)	−0.0054 (10)	0.0009 (9)	0.0153 (11)
C11	0.0422 (14)	0.0530 (17)	0.0480 (15)	−0.0059 (13)	0.0102 (12)	0.0031 (13)
C12	0.0480 (14)	0.0453 (16)	0.0459 (15)	−0.0115 (12)	0.0102 (12)	−0.0005 (12)
C13	0.0420 (14)	0.0526 (17)	0.0427 (14)	−0.0059 (13)	0.0084 (11)	0.0005 (12)
C14	0.0469 (14)	0.0473 (17)	0.0482 (15)	−0.0036 (13)	0.0138 (12)	0.0015 (12)
C15	0.0509 (16)	0.0509 (17)	0.0643 (18)	−0.0130 (14)	0.0171 (14)	−0.0060 (14)
C16	0.0418 (14)	0.062 (2)	0.0598 (18)	−0.0137 (14)	0.0092 (13)	−0.0027 (15)
O17	0.0470 (10)	0.0523 (12)	0.0707 (13)	−0.0082 (9)	0.0010 (9)	0.0051 (10)
C18	0.0486 (16)	0.061 (2)	0.078 (2)	−0.0199 (14)	0.0042 (14)	0.0031 (16)
O19	0.0563 (11)	0.0441 (12)	0.0732 (13)	−0.0037 (9)	0.0138 (10)	−0.0016 (9)
C20	0.0727 (19)	0.0473 (18)	0.081 (2)	−0.0054 (16)	0.0270 (17)	−0.0061 (16)

### Geometric parameters (Å, °)

**Table:**

F1—C3	1.343 (3)	C11—C12	1.398 (3)
F2—C4	1.354 (3)	C12—C13	1.372 (4)
C1—C6	1.375 (4)	C12—H12	0.93
C1—C2	1.382 (3)	C13—O17	1.369 (3)
C1—N7	1.434 (4)	C13—C14	1.405 (4)
C2—C3	1.357 (4)	C14—O19	1.350 (3)
C2—H2	0.93	C14—C15	1.379 (3)
C3—C4	1.359 (4)	C15—C16	1.378 (4)
C4—C5	1.352 (4)	C15—H15	0.93
C5—C6	1.383 (4)	C16—H16	0.93
C5—H5	0.93	O17—C18	1.420 (3)
C6—H6	0.93	C18—H18A	0.96
N7—S8	1.628 (3)	C18—H18B	0.96
N7—H7	0.76 (3)	C18—H18C	0.96
S8—O10	1.424 (2)	O19—C20	1.431 (3)
S8—O9	1.424 (2)	C20—H20A	0.96
S8—C11	1.754 (3)	C20—H20B	0.96
C11—C16	1.381 (4)	C20—H20C	0.96
			
C6—C1—C2	119.3 (3)	C13—C12—C11	119.6 (2)
C6—C1—N7	119.9 (2)	C13—C12—H12	120.2
C2—C1—N7	120.7 (3)	C11—C12—H12	120.2
C3—C2—C1	119.2 (3)	O17—C13—C12	125.1 (2)
C3—C2—H2	120.4	O17—C13—C14	114.6 (2)
C1—C2—H2	120.4	C12—C13—C14	120.3 (2)
F1—C3—C2	120.1 (3)	O19—C14—C15	125.5 (2)
F1—C3—C4	118.5 (3)	O19—C14—C13	114.9 (2)
C2—C3—C4	121.4 (3)	C15—C14—C13	119.6 (3)
C5—C4—F2	120.7 (3)	C16—C15—C14	120.1 (3)
C5—C4—C3	120.4 (3)	C16—C15—H15	119.9
F2—C4—C3	118.9 (3)	C14—C15—H15	119.9
C4—C5—C6	119.3 (3)	C15—C16—C11	120.5 (2)
C4—C5—H5	120.3	C15—C16—H16	119.8
C6—C5—H5	120.3	C11—C16—H16	119.8
C1—C6—C5	120.3 (3)	C13—O17—C18	118.3 (2)
C1—C6—H6	119.8	O17—C18—H18A	109.5
C5—C6—H6	119.8	O17—C18—H18B	109.5
C1—N7—S8	123.2 (2)	H18A—C18—H18B	109.5
C1—N7—H7	109 (2)	O17—C18—H18C	109.5
S8—N7—H7	115 (2)	H18A—C18—H18C	109.5
O10—S8—O9	120.03 (13)	H18B—C18—H18C	109.5
O10—S8—N7	107.04 (13)	C14—O19—C20	117.3 (2)
O9—S8—N7	105.36 (14)	O19—C20—H20A	109.5
O10—S8—C11	107.51 (13)	O19—C20—H20B	109.5
O9—S8—C11	108.15 (12)	H20A—C20—H20B	109.5
N7—S8—C11	108.29 (13)	O19—C20—H20C	109.5
C16—C11—C12	119.9 (2)	H20A—C20—H20C	109.5
C16—C11—S8	120.0 (2)	H20B—C20—H20C	109.5
C12—C11—S8	120.0 (2)		

### Hydrogen-bond geometry (Å, °)

**Table:**

*D*—H···*A*	*D*—H	H···*A*	*D*···*A*	*D*—H···*A*
N7—H7···O17^i^	0.76 (3)	2.48 (3)	3.180 (3)	155 (3)
N7—H7···O19^i^	0.76 (3)	2.61 (3)	3.256 (3)	144 (3)

Symmetry codes: (i) −*x*+2, *y*+1/2, −*z*+1/2.
